# Supplementary material for: A semantic segmentation model to predict subcellular glycogen localization using transmission electron microscopy images
Source: PLoS One. 2026 Jun 17;21(6):e0351502. doi: 10.1371/journal.pone.0351502 (PMC13274865; doi:10.1371/journal.pone.0351502)
Supplement: Attachment — Submitted filename: Response to Reviewers.docx [file pone.0351502.s001.docx]

**We thank the reviewers for their thoughtful comments and constructive suggestions. We have addressed all comments point-by-point below and believe that the revisions have improved the clarity and quality of the manuscript.**

Reviewer #1: This is a very interesting and potentially useful methodological paper addressing an important barrier in the study of subcellular glycogen localisation in skeletal muscle. The topic is relevant, the manuscript is generally clear, and the open sharing of code, data, and model weights is a strength. However, I have several concerns that should be addressed before the manuscript can be considered further.

Overall, my main concern is that some of the conclusions appear stronger than the validation presented. The study shows promising agreement with manual image analysis, but there are important limitations related to the reference standard, the treatment of subsarcolemmal images, and the independence and size of the training and testing datasets.

Major comments

The manuscript presents the model as validated, robust, and objective. However, the reference standard appears to rely on manual annotation and point counting by a single experienced investigator. This demonstrates agreement with one expert, but not necessarily broader validity or reproducibility. Do authors have any data on how results differ between different experienced researchers?

**Reply:**

**In this project, the model output was compared with analyses performed by a single experienced researcher (J.N.). The same researcher has led previous studies in which point-counting data from multiple researchers were compared. In these studies, inter-researcher bias reached up to 23% (e.g. Schytz et al., 2024). The bias observed for the model is therefore within the range of biases previously reported between experienced researchers.**

**We agree that the robustness of the model across different researchers has not been formally investigated. In the manuscript, the term “robust” was intended to refer to the model’s performance across different glycogen levels; however, we acknowledge that this was unclear in the abstract. Accordingly, we have added the following sentence (line 313-315):**

**“The level of bias (10–15%) is within the range of biases observed between different researchers (e.g. Schytz et al., 2024). We therefore interpret the model as a valid substitute for researcher-based manual analyses.”**

**In addition, we have removed the term “robust” from line 32.**

A major concern is that the region model was trained on myofibrillar images, but then applied to subsarcolemmal images, with the intermyofibrillar class subsequently relabelled as subsarcolemmal. This appears to be an ad hoc solution and needs much stronger justification. If subsarcolemmal glycogen is a key part of the paper, this limitation should be more explicitly acknowledged and discussed.

**Reply:**

**Due to the high magnification used, the image area was relatively small and contained predominantly subsarcolemmal glycogen. Therefore, during model development, we considered that the model’s ability to identify glycogen would likely be sufficient for estimating glycogen content within the subsarcolemmal region, despite not being specifically trained to distinguish subsarcolemmal from intermyofibrillar regions. The validation results support this assumption.**

**We agree with the reviewer that this limitation should be more explicitly acknowledged and discussed in the manuscript. Therefore, we have added the following paragraph to the Discussion (line 316-321):**

**“A limitation of the model is that it was not trained to discriminate between subsarcolemmal and intermyofibrillar regions. However, in most images classified as subsarcolemmal, contamination from the intermyofibrillar region was minimal due to the small image size (1.7 × 1.7 µm), in which the subsarcolemmal region occupied the majority of the image area. This may be specific to human muscle samples, and additional caution may therefore be required when applying the model to muscles with fewer subsarcolemmal organelles and inclusions.”**

The rationale for the number of samples used for model development and evaluation should be explained more clearly. For example, in lines 108 to 116, images were obtained from 12 biopsies from 4 participants for manual segmentation and training, while all 21 biopsies were used for the biochemical comparison. Please explain how these numbers were determined and whether the authors believe that inclusion of more biopsies, fibres, or participants would further improve model performance or generalisability. This is particularly relevant for a machine learning based approach.

**Reply:**

**The biopsies originated from a study including three time points with known large variability in glycogen content. These biopsies were therefore considered suitable for developing a model capable of handling a broad range of glycogen levels. To capture this variability, images were obtained from all three time points.**

**Initially, 429 images from two participants were manually annotated and used for model training. However, the resulting performance was considered insufficient for accurate estimation. Therefore, an additional 430 images from three other participants were annotated and included in the training dataset. No formal analysis was performed to determine how model performance scales with increasing numbers of biopsies, fibres, or participants, and we agree that inclusion of additional data could potentially further improve both model performance and generalisability.**

**To clarify this process, we have added information regarding these two training steps to the Methods section (lines 144–146): "For initial training 429 images from two participants were annotated but considered inadequate for accurate estimates. Therefore, additional 430 images from three other participants were annotated."**

**The biochemical comparison was included as a secondary validation in addition to the comparison with manually annotated ground truth data. Therefore, this analysis was performed using model predictions from all available biopsies (n = 21).**

The results suggest systematic bias, with underestimation in intermyofibrillar and subsarcolemmal glycogen and overestimation in intramyofibrillar glycogen. This is an important limitation because the intended application of the model is compartment specific analysis. The discussion does acknowledge this, but I think the conclusion should be more cautious. Or, there should be some justified reassurance that this under/overestimation is small enough to still make the model valid.

**Reply:**

**We agree that the observed systematic underestimation and overestimation across glycogen compartments represent an important consideration for compartment-specific analyses. However, we interpret these biases as sufficiently small to support the validity of the model, as they fall within the range of biases previously observed between experienced researchers performing manual analyses.**

**To clarify this justification, we have added the following statement to the Discussion (lines 313–315):**

**“The level of bias (10–15%) is within the range of biases observed between different researchers (e.g. Schytz et al., 2024). We therefore interpret the model as a valid substitute for researcher-based manual analyses.”**

Last but not least - are authors confident that the presented model is sufficiently accurate to be used in further research by other scientists?

**Reply:**

**We believe the presented model is sufficiently accurate to serve as a useful tool for future research applications. However, we also expect that optimal performance in new projects or datasets may require project-specific fine-tuning and validation.**

**To clarify this limitation, we have added the following statement to the Discussion (line 312):**

**“…and most likely a need for fine-tuning of the model.”**

Minor comments
Line 49 - “Therefor” should be corrected to “therefore.”

**Reply:**

**Done, thanks.**

Lines 50 to 52 - It would help the reader if the authors briefly explained why analysis of 8 to 10 fibres per biopsy is typically needed. At present, this appears without much context. Since later results show that uncertainty decreases as more images are included, it would be useful to link this statement to the practical sampling considerations for biopsy level estimates.

**Reply:**

**We agree that additional context was needed. There is currently no formal documentation establishing that 8–10 fibres are specifically required for reliable biopsy-level estimates. Rather, this number reflects a pragmatic balance between the time-consuming nature of the method and the well-known inter-fibre variability in glycogen content, where individual fibre values commonly vary within an approximately threefold range.**

**To clarify this point, we have modified the sentence in line 52 to read:**

**“…which is typically included in such studies.”**

Lines 81 to 82 - Please provide a little more detail on what is meant by “samples were processed for transmission electron microscopy.” Even a brief summary of the key preparation steps would improve clarity for readers who are less familiar with TEM workflows, or a reference to a paper describing them in details.

**Reply:**

**To clarify this, we now refer to the later section describing the TEM workflow. We have added (line 85): "(see fixation procedure below)".**

Lines 84-92 - Authors state that two methods were used for biochemical method of glycogen quantification. However, it is unclear results of which method were subsequently used in the results section.

**Reply:**

**We agree that this was unclear in the original manuscript. Therefore, we have added the following sentence (lines 97–101):**

**“The data used for comparison with TEM analyses were obtained using the Pentra C400 analyser for the pre-exercise and recovery samples, and the Fluoroskan instrument for the post-exercise samples.** **The Fluoroskan instrument has higher sensitivity for glycogen measurements at very low concentrations.”**

Lines 90 to 92 Although this appears in the Methods, the phrase “unexpectedly low glycogen values” would benefit from a little more context. Unexpected relative to what, previous work, biological expectation, or the second assay? A short explanation would help readers understand why the second analytical approach was needed.

**Reply:**

**We agree that additional context was needed. In fact, the term “unexpectedly low” was not appropriate, as the exercise protocol was identical to that used by Bergström and Hultman (1966), who reported similarly low glycogen levels. Therefore, we have replaced “unexpectedly low” with “very low.”**

**In addition, we have clarified why the second analytical approach was used by adding the following text (lines 97–101):**

**“The data used for comparison with TEM analyses were obtained using the Pentra C400 analyser for the pre-exercise and recovery samples, and the Fluoroskan instrument for the post-exercise samples. The Fluoroskan instrument has higher sensitivity for glycogen measurements at very low concentrations.”**

Lines 108 to 116 - As noted above, please explain the reasoning behind the number of biopsies, participants, and fibres used for model training and validation. It would also be helpful to comment on whether larger training input might improve performance.

**Reply:**

**The rationale for the two-step training procedure has now been clarified in lines 144–146: "For initial training 429 images from two participants were annotated but considered inadequate for accurate estimates. Therefore, additional 430 images from three other participants were annotated."**

**In addition, we have added the following explanation regarding the number of fibres analysed per biopsy (line 125-127):**

**“This number of fibres per biopsy has previously been shown to provide TEM-based estimates of total glycogen that are proportional to values obtained by biochemical determination (e.g. Schytz et al., 2024).”**

**We also acknowledge that increasing the number of biopsies, fibres, and participants included in the training dataset could potentially improve model performance and generalisability, although this was not formally investigated in the present study.**

Reviewer #2: The authors developed a semantic segmentation model for glycogen particles in transmission electron microscopy images of skeletal muscle. Due to the time-consuming nature of manual analyses of glycogen particles and the critical role of glycogen in muscle metabolism, faster analytical methods are much needed. The developed model appears to work well. However, it would be helpful to better distinguish this model from a recently developed, similar glycogen model.

I appreciate that the authors are clear that their approach is similar to another recent work (Rios et al. J Gen Physiol 2024). However, it is not as clear why they developed a second approach instead of just adopting the original one. The authors state that the new model can better distinguish Z-disks and mitochondria than the previous model, and thus implied that this model is better equipped to assess fibers with different Z-disk widths and mitochondrial content. Demonstrating that the model performs well in different muscle fiber types would support these statements. Based on their extensive history of subcellular glycogen quantification, the authors likely have many ground truth images from different fiber types to compare with model output. Given that the code for the Rios et al. model appears to be freely available, and even better approach, but perhaps not necessary, would be to compare the current model versus the original model.

**Reply:**

**The development of our model was initiated before the code from the study by Eduardo Ríos et al. became publicly available. Once the code was released, we contacted Eduardo Ríos to discuss potential directions for the project.**

**A key objective of our model development was the ability to distinguish between Z-disks and mitochondria, as this would enable future studies investigating glycogen localization in relation to mitochondrial volume fraction. For this reason, we continued the development of our own model rather than directly adopting the existing approach.**

**We agree that demonstrating performance across different muscle fibre types would strengthen this point. However, in our previous studies, glycogen content was quantified using point-counting methods to estimate glycogen areal fractions, and therefore no pixel-level ground truth annotations are available from those datasets for direct validation of the present model.**

**Reviewer #3 raised a similar question regarding comparison with the model by Rios et al., and we therefore refer to our response to Reviewer #3 for additional details on this point.**

Since the major error seems to be in region segmentation rather than glycogen segmentation, it would be helpful to see the ground truth segmentations included in the panels in Figures 1 and 2.

**Reply:**

**The ground truth has been added to Figures 1 and 2.**

Figure 1: What do the different colors represent in the Predicted Glycogen panels? It is not clear from the figure, legend, or text. If it is glycogen probabilities as it looks to be, it would be helpful to also include the binary glycogen images (using probability threshold of 0.3 as stated in methods) to make it clear which glycogen particles are counted and which are not.

**Reply:**

**We agree. We have changed the predictions to be the binary glycogen images at threshold 0.3.**


Minor Comments
Methods, Participants and Muscle Samples: Which biopsy method was used?

**Reply:**

**Added to line 82: "(using the Bergström needle with suction)"**

Reviewer #3: The paper tackles a challenging problem with a well-motivated approach. However, the clarity of the presentation needs improvement, making it difficult to fully appreciate the paper's contributions.

Bellow, I list specific points that could be addressed to enhance the clarity and impact of the paper.

The relationship to the parallel approach of Rios et al. is unclear. Lines 55-62 attempt to address this, but the wording is vague. The use of past tense ("we aimed to develop") and the mention of Rios's model being available raise doubts about whether your model was developed as intended. It is also unclear whether you are arguing for the advantages of your model over Rios's, or simply listing features of yours model.

**Reply:**

**We initiated development of our model before the release of the model by Eduardo Ríos et al. After their model became publicly available, we contacted Eduardo Ríos to clarify several methodological details.**

**We included their work in the Introduction to be fully transparent that a related model already exists, although our own model was developed largely independently and was only minimally influenced by their approach during the later stages of development.**

**We agree that the original wording did not clearly distinguish whether we were describing advantages of our model or simply listing its features. Our preference for the present model is primarily related to its ability to distinguish mitochondria, which we consider important for future studies investigating glycogen localization in relation to mitochondrial content. At the same time, we acknowledge that the model by Rios et al. includes features that are not part of our model.**

**Therefore, to provide a more balanced comparison between the two approaches, we have added the following sentence (lines 63–65):**

**“On the other hand, their model includes regions of sarcoplasmic reticulum and near-sarcoplasmic reticulum, which are not included in our model.”**


The connection to the larger project involving this data is also ambiguous. For example, in lines 74-75, you state that biopsies have varying glycogen content, but it is not clear how this was established. If this is based on methods described in lines 87-92, the order of presentation is confusing. The mention of unexpectedly low values (line 90) lacks context—what are these values, and were they confirmed by second method?

**Reply:**

**We agree that the connection between the TEM model development and the larger project was not sufficiently clear, and that the order of presentation may therefore have been confusing. The variability in glycogen content between biopsies had already been established from the biochemical analyses before initiation of the TEM model development.**

**To clarify this, we have added the following sentence (lines 86–87):**

**“At the initiation of the development of the TEM model, biochemical measurements of glycogen content were already available.”**

**In addition, we have clarified the description of the very low glycogen values and the use of the second analytical method (lines 97–101), including that these low values were confirmed using a more sensitive assay.**


Details about manual annotation for training or testing are scattered throughout the paper. It would be helpful to clearly summarize the data used: the number of images, the number of annotations, and what exactly was annotated (label images or point-counting). The extent of training data for the glycogen segmentation model is unclear.

**Reply:**

**We agree that the details regarding manual annotation and validation data were scattered throughout the manuscript and therefore difficult to follow. To improve clarity, we have added the following summary paragraph (lines 197–206):**

**“Summary of datasets used for training and validation. The training dataset for the region model consisted of 859 images that were partially annotated for five labels: Z-disc, I-band, A-band, mitochondria, and intermyofibrillar region. For testing the accuracy of the region model, 92 images were fully annotated for all five labels.**

**The training dataset for the glycogen model consisted of 100 images that were partially annotated for two labels: glycogen and non-glycogen. For testing the accuracy of the glycogen model, glycogen areal fraction was estimated in the same 92 images using point-counting.**

**Validation of the combined model was performed using these 92 images, in which glycogen areal fractions within the intramyofibrillar and intermyofibrillar regions were estimated by point-counting. In addition, glycogen areal fraction within the subsarcolemmal region was estimated by point-counting in 84 images.”**

Several methodological choices are not explained or justified. Why use two unrelated segmentation models? Would it be beneficial to share parts of the segmentation networks? If the glycogen model's predictions are used to extract particles, why not train the model to directly detect particles?

**Reply:**

**We acknowledge that shared architectures can be beneficial in multi-task settings, particularly when tasks share low-level features or benefit from inductive transfer. In this case, however, we opted for two separate models for several reasons. Decoupling the tasks allowed us to annotate each independently, which was a substantial practical advantage. A shared model would require region annotations that carefully exclude glycogen particles from the surrounding fiber regions and would substantially increase the annotation burden. Separate models also enable validation with task-appropriate metrics and direct comparison against established methods during development, such as the point-based quantification for the glycogen model. Beyond practical considerations, it is not clear that features learned for fiber-type segmentation would aid glycogen detection, since a glycogen particle has the same local appearance regardless of its location within a fiber. Separation further allows us to match capacity to task complexity, using a smaller glycogen model to reduce overfitting risk and an additional layer in the fiber-region model to handle its more complex problem. Finally, it gives us the freedom to choose different loss functions and data preprocessing and augmentation procedures. We have added this to the discussion (line 335-341):**

**"The present approach used two separate segmentation models rather than a shared multi-task architecture. This design was chosen to allow independent annotation and task-specific optimization, which substantially reduced annotation complexity and enabled validation against established methods. In addition, it remains unclear whether features learned for fibre-region segmentation would improve glycogen detection, as glycogen particles exhibit similar local appearance across fibre regions. Future studies could investigate whether shared architectures or direct particle-detection approaches may further improve performance and efficiency."**

**Regarding direct particle detection, we agree that this may represent a more optimal approach than the current extraction procedure based on glycogen segmentation. However, such an approach was beyond the scope of the present study. To acknowledge this limitation and future possibility, we have added the following statement to the Discussion (line 297-300):**

**“However, it would be relevant to investigate whether a model could be trained to directly identify individual particles within glycogen clusters, thereby avoiding assumptions regarding particle shape during extraction of particle size.”**


Why is the region model trained only on the myofibrillar compartment but used also on subsarcolemmal data. The explanation in lines 149-159 is awkward, and the sentence in line 154 about subsarcolemmal images appears too late. This highlights the need for a thorough explanation of the data used.

**Reply:**

**We agree that the original explanation was unclear and that the rationale for applying the region model to subsarcolemmal images needed to be described more explicitly. Therefore, we have revised the paragraph as follows (line 162-167):**

**“Although the model was trained on myofibrillar images, it performed adequately on subsarcolemmal images, where it reliably identified glycogen. In this context, predictions assigned to the intermyofibrillar class were interpreted as subsarcolemmal, while the remaining structural classes were combined into a non-subsarcolemmal (Non-SS) category. This interpretation was supported by the imaging approach, which minimized inclusion of intermyofibrillar regions within the subsarcolemmal images.”**


When discussing model accuracy in line 160, it is unclear whether the 92 images refer to myofibrillar, subsarcolemmal, or both types.

**Reply:**

**We have added "... myofibrillar images"**


It is ambiguous what you mean by "model evaluation". Are you describing the process of model validation (e.g., hyperparameter selection) or reporting the final performance.

**Reply:**

**We have changed the wording in the abstract and method line 187 to be more precise.**


On a higher level, it is unclear whether you are proposing an approach for others to follow or offering a model for direct use. Clarifying the intended benefits and the types of problems your work addresses would help the community understand its value.

**Reply:**

**We agree that the intended use of the work should be clarified more explicitly. We primarily view the present study as providing an approach that other researchers can adapt and build upon for automated TEM-based glycogen analysis. However, the model may also be directly applicable in studies using similar sample preparation procedures and image acquisition settings.**

**To clarify this point, we have added the following statement to the Discussion section (lines 331–334):**

**“Using the same fixation protocol and image resolution, the model can most likely be applied directly. However, under different experimental or imaging conditions, fine-tuning of the model prior to use may be necessary.”**


The figures could be clearer. For example, say "predictions of the region model" or "predicted regions" rather than "predicted locations". Consider showing ground truth annotations. Yellow glycogen overlays are not visible on top of yellow locations. The confusion matrices in Figure 3 have six categories, but the model predicts only five, making interpretation difficult. The numbers in parentheses are explained, but remain unclear. Results for the three intra-compartments are sparse, despite three regions being a main motivation for this work and were listed as advantage. Consider presenting these results more explicitly. Why not show confusion matrix for glycogen segmentation model?

**Reply:**

**We thank the reviewer for these constructive suggestions regarding the figures and presentation of the results.**

**In Figures 1 and 2, we have changed the terminology from “predicted locations” to “predicted regions,” included examples of the ground truth annotations, and adjusted the colors of the Z-disc and glycogen overlays to improve visibility.**

**In Figure 3, we have added an explanatory sentence below the confusion matrix to clarify the numbers shown in parentheses. In addition, the extra “intra” region is now explicitly explained in the figure legend (lines 455–456).**

**We agree that a confusion matrix for the glycogen segmentation model would have been valuable. However, this model was validated using comparisons between predicted glycogen content and glycogen areal fraction estimated by point-counting, rather than pixel-level glycogen annotations. Therefore, a conventional confusion matrix could not be generated for this model.**

**Regarding the intramyofibrillar subcompartments (I-band and A-band), the purpose of including these labels was primarily to demonstrate the flexibility and future potential of the region model. In our previous studies, we have intentionally not separated glycogen content between I-band and A-band regions because the biopsy procedure does not standardize fibre length. This introduces substantial variability in sarcomere length and, consequently, in the relative areal fraction of the I-band between samples. Due to the isovolumetric nature of muscle fibers, variability in sarcomere length does not affect glycogen particle density within the combined intramyofibrillar compartment. Therefore, in the present manuscript, we chose to focus on the combined intramyofibrillar glycogen measure while still documenting the region model’s ability to distinguish I-band and A-band structures.**
