# Supplementary material for: A semantic segmentation model to predict subcellular glycogen localization using transmission electron microscopy images
Source: PLoS One. 2026 Jun 17;21(6):e0351502. doi: 10.1371/journal.pone.0351502 (PMC13274865; doi:10.1371/journal.pone.0351502)
Supplement: Peer review history

# Peer review: Decision Letter 0

**DOI:** 10.1371/journal.pone.0351502.r001

**Authors:** Everson Nunes

1 Apr 2026

PONE-D-26-06780

A semantic segmentation model to predict subcellular glycogen localization using transmission electron microscopy images

PLOS One

Dear Dr. Nielsen,

Thank you for submitting your manuscript to PLOS ONE. After careful consideration, we feel that it has merit but does not fully meet PLOS ONE’s publication criteria as it currently stands. Therefore, we invite you to submit a revised version of the manuscript that addresses the points raised during the review process.

Please submit your revised manuscript by May 16 2026 11:59PM. If you will need more time than this to complete your revisions, please reply to this message or contact the journal office at plosone@plos.org. When you're ready to submit your revision, log on to https://www.editorialmanager.com/pone/ and select the 'Submissions Needing Revision' folder to locate your manuscript file.

Please include the following items when submitting your revised manuscript:

- A letter that responds to each point raised by the academic editor and reviewer(s). You should upload this letter as a separate file labeled 'Response to Reviewers'.
- A marked-up copy of your manuscript that highlights changes made to the original version. You should upload this as a separate file labeled 'Revised Manuscript with Track Changes'.
- An unmarked version of your revised paper without tracked changes. You should upload this as a separate file labeled 'Manuscript'.

If you would like to make changes to your financial disclosure, please include your updated statement in your cover letter. Guidelines for resubmitting your figure files are available below the reviewer comments at the end of this letter.

If applicable, we recommend that you deposit your laboratory protocols in protocols.io to enhance the reproducibility of your results. Protocols.io assigns your protocol its own identifier (DOI) so that it can be cited independently in the future. For instructions see: https://journals.plos.org/plosone/s/submission-guidelines#loc-laboratory-protocols. Additionally, PLOS ONE offers an option for publishing peer-reviewed Lab Protocol articles, which describe protocols hosted on protocols.io. Read more information on sharing protocols at https://plos.org/protocols?utm_medium=editorial-email&utm_source=authorletters&utm_campaign=protocols.

As the corresponding author, your ORCID iD is verified in the submission system and will appear in the published article. PLOS supports the use of ORCID, and we encourage all coauthors to register for an ORCID iD and use it as well. Please encourage your coauthors to verify their ORCID iD within the submission system before final acceptance, as unverified ORCID iDs will not appear in the published article. *Only*   the individual author can complete the verification step; PLOS staff *cannot*   verify ORCID iDs on behalf of authors.

We look forward to receiving your revised manuscript.

Kind regards,

Everson Nunes, Ph.D.

Academic Editor

PLOS One

Journal Requirements:

When submitting your revision, we need you to address these additional requirements.

1.Please ensure that your manuscript meets PLOS ONE's style requirements, including those for file naming. The PLOS ONE style templates can be found at

https://journals.plos.org/plosone/s/file?id=wjVg/PLOSOne_formatting_sample_main_body.pdf and

https://journals.plos.org/plosone/s/file?id=ba62/PLOSOne_formatting_sample_title_authors_affiliations.pdf

2. Please note that funding information should not appear in any section or other areas of your manuscript. We will only publish funding information present in the Funding Statement section of the online submission form. Please remove any funding-related text from the manuscript.

3. Thank you for stating the following financial disclosure:

“This work was funded by The Independent Research Fund Denmark (grant 10.46540/3103-00131B to JN).”

Please state what role the funders took in the study.  If the funders had no role, please state: "The funders had no role in study design, data collection and analysis, decision to publish, or preparation of the manuscript."

If this statement is not correct you must amend it as needed.

Please include this amended Role of Funder statement in your cover letter; we will change the online submission form on your behalf.

4. Please note that your Data Availability Statement is currently missing the repository name. If your manuscript is accepted for publication, you will be asked to provide these details on a very short timeline. We therefore suggest that you provide this information now, though we will not hold up the peer review process if you are unable.

5. If the reviewer comments include a recommendation to cite specific previously published works, please review and evaluate these publications to determine whether they are relevant and should be cited. There is no requirement to cite these works unless the editor has indicated otherwise.

Additional Editor Comments:

Dear authors,

The manuscript has now been evaluated by three independent reviewers. Their reports are generally aligned in identifying the study’s relevance and potential contribution, but they also raise some points that need to be addressed before the manuscript can be considered further.

[Note: HTML markup is below. Please do not edit.]

Reviewers' comments:

Reviewer's Responses to Questions

**Comments to the Author**

1. Is the manuscript technically sound, and do the data support the conclusions?

The manuscript must describe a technically sound piece of scientific research with data that supports the conclusions. Experiments must have been conducted rigorously, with appropriate controls, replication, and sample sizes. The conclusions must be drawn appropriately based on the data presented.

Reviewer #1: Yes

Reviewer #2: Yes

Reviewer #3: Yes

**********

2. Has the statistical analysis been performed appropriately and rigorously?

Reviewer #1: Yes

Reviewer #2: Yes

Reviewer #3: Yes

**********

3. Have the authors made all data underlying the findings in their manuscript fully available?

The PLOS Data policy requires authors to make all data underlying the findings described in their manuscript fully available without restriction, with rare exception (please refer to the Data Availability Statement in the manuscript PDF file). The data should be provided as part of the manuscript or its supporting information, or deposited to a public repository. For example, in addition to summary statistics, the data points behind means, medians and variance measures should be available. If there are restrictions on publicly sharing data—e.g. participant privacy or use of data from a third party—those must be specified.

Reviewer #1: Yes

Reviewer #2: Yes

Reviewer #3: Yes

**********

4. Is the manuscript presented in an intelligible fashion and written in standard English?

PLOS ONE does not copyedit accepted manuscripts, so the language in submitted articles must be clear, correct, and unambiguous. Any typographical or grammatical errors should be corrected at revision, so please note any specific errors here.

Reviewer #1: Yes

Reviewer #2: Yes

Reviewer #3: No

**********

5. Review Comments to the Author

Please use the space provided to explain your answers to the questions above. You may also include additional comments for the author, including concerns about dual publication, research ethics, or publication ethics. (Please upload your review as an attachment if it exceeds 20,000 characters)

Reviewer #1: This is a very interesting and potentially useful methodological paper addressing an important barrier in the study of subcellular glycogen localisation in skeletal muscle. The topic is relevant, the manuscript is generally clear, and the open sharing of code, data, and model weights is a strength. However, I have several concerns that should be addressed before the manuscript can be considered further.

Overall, my main concern is that some of the conclusions appear stronger than the validation presented. The study shows promising agreement with manual image analysis, but there are important limitations related to the reference standard, the treatment of subsarcolemmal images, and the independence and size of the training and testing datasets.

Major comments

The manuscript presents the model as validated, robust, and objective. However, the reference standard appears to rely on manual annotation and point counting by a single experienced investigator. This demonstrates agreement with one expert, but not necessarily broader validity or reproducibility. Do authors have any data on how results differ between different experienced researchers?

A major concern is that the region model was trained on myofibrillar images, but then applied to subsarcolemmal images, with the intermyofibrillar class subsequently relabelled as subsarcolemmal. This appears to be an ad hoc solution and needs much stronger justification. If subsarcolemmal glycogen is a key part of the paper, this limitation should be more explicitly acknowledged and discussed.

The rationale for the number of samples used for model development and evaluation should be explained more clearly. For example, in lines 108 to 116, images were obtained from 12 biopsies from 4 participants for manual segmentation and training, while all 21 biopsies were used for the biochemical comparison. Please explain how these numbers were determined and whether the authors believe that inclusion of more biopsies, fibres, or participants would further improve model performance or generalisability. This is particularly relevant for a machine learning based approach.

The results suggest systematic bias, with underestimation in intermyofibrillar and subsarcolemmal glycogen and overestimation in intramyofibrillar glycogen. This is an important limitation because the intended application of the model is compartment specific analysis. The discussion does acknowledge this, but I think the conclusion should be more cautious. Or, there should be some justified reassurance that this under/overestimation is small enough to still make the model valid.

Last but not least - are authors confident that the presented model is sufficiently accurate to be used in further research by other scientists?

Minor comments

Line 49 - “Therefor” should be corrected to “therefore.”

Lines 50 to 52 - It would help the reader if the authors briefly explained why analysis of 8 to 10 fibres per biopsy is typically needed. At present, this appears without much context. Since later results show that uncertainty decreases as more images are included, it would be useful to link this statement to the practical sampling considerations for biopsy level estimates.

Lines 81 to 82 - Please provide a little more detail on what is meant by “samples were processed for transmission electron microscopy.” Even a brief summary of the key preparation steps would improve clarity for readers who are less familiar with TEM workflows, or a reference to a paper describing them in details.

Lines 84-92 - Authors state that two methods were used for biochemical method of glycogen quantification. However, it is unclear results of which method were subsequently used in the results section.

Lines 90 to 92 Although this appears in the Methods, the phrase “unexpectedly low glycogen values” would benefit from a little more context. Unexpected relative to what, previous work, biological expectation, or the second assay? A short explanation would help readers understand why the second analytical approach was needed.

Lines 108 to 116 - As noted above, please explain the reasoning behind the number of biopsies, participants, and fibres used for model training and validation. It would also be helpful to comment on whether larger training input might improve performance.

Reviewer #2: The authors developed a semantic segmentation model for glycogen particles in transmission electron microscopy images of skeletal muscle. Due to the time-consuming nature of manual analyses of glycogen particles and the critical role of glycogen in muscle metabolism, faster analytical methods are much needed. The developed model appears to work well. However, it would be helpful to better distinguish this model from a recently developed, similar glycogen model.

I appreciate that the authors are clear that their approach is similar to another recent work (Rios et al. J Gen Physiol 2024). However, it is not as clear why they developed a second approach instead of just adopting the original one. The authors state that the new model can better distinguish Z-disks and mitochondria than the previous model, and thus implied that this model is better equipped to assess fibers with different Z-disk widths and mitochondrial content. Demonstrating that the model performs well in different muscle fiber types would support these statements. Based on their extensive history of subcellular glycogen quantification, the authors likely have many ground truth images from different fiber types to compare with model output. Given that the code for the Rios et al. model appears to be freely available, and even better approach, but perhaps not necessary, would be to compare the current model versus the original model.

Since the major error seems to be in region segmentation rather than glycogen segmentation, it would be helpful to see the ground truth segmentations included in the panels in Figures 1 and 2.

Figure 1: What do the different colors represent in the Predicted Glycogen panels? It is not clear from the figure, legend, or text. If it is glycogen probabilities as it looks to be, it would be helpful to also include the binary glycogen images (using probability threshold of 0.3 as stated in methods) to make it clear which glycogen particles are counted and which are not.

Minor Comments

Methods, Participants and Muscle Samples: Which biopsy method was used?

Reviewer #3: The paper tackles a challenging problem with a well-motivated approach. However, the clarity of the presentation needs improvement, making it difficult to fully appreciate the paper's contributions.

Bellow, I list specific points that could be addressed to enhance the clarity and impact of the paper.

The relationship to the parallel approach of Rios et al. is unclear. Lines 55-62 attempt to address this, but the wording is vague. The use of past tense ("we aimed to develop") and the mention of Rios's model being available raise doubts about whether your model was developed as intended. It is also unclear whether you are arguing for the advantages of your model over Rios's, or simply listing features of yours model.

The connection to the larger project involving this data is also ambiguous. For example, in lines 74-75, you state that biopsies have varying glycogen content, but it is not clear how this was established. If this is based on methods described in lines 87-92, the order of presentation is confusing. The mention of unexpectedly low values (line 90) lacks context—what are these values, and were they confirmed by second method?

Details about manual annotation for training or testing are scattered throughout the paper. It would be helpful to clearly summarize the data used: the number of images, the number of annotations, and what exactly was annotated (label images or point-counting). The extent of training data for the glycogen segmentation model is unclear.

Several methodological choices are not explained or justified. Why use two unrelated segmentation models? Would it be beneficial to share parts of the segmentation networks? If the glycogen model's predictions are used to extract particles, why not train the model to directly detect particles?

Why is the region model trained only on the myofibrillar compartment but used also on subsarcolemmal data. The explanation in lines 149-159 is awkward, and the sentence in line 154 about subsarcolemmal images appears too late. This highlights the need for a thorough explanation of the data used.

When discussing model accuracy in line 160, it is unclear whether the 92 images refer to myofibrillar, subsarcolemmal, or both types.

It is ambiguous what you mean by "model evaluation". Are you describing the process of model validation (e.g., hyperparameter selection) or reporting the final performance.

On a higher level, it is unclear whether you are proposing an approach for others to follow or offering a model for direct use. Clarifying the intended benefits and the types of problems your work addresses would help the community understand its value.

The figures could be clearer. For example, say "predictions of the region model" or "predicted regions" rather than "predicted locations". Consider showing ground truth annotations. Yellow glycogen overlays are not visible on top of yellow locations. The confusion matrices in Figure 3 have six categories, but the model predicts only five, making interpretation difficult. The numbers in parentheses are explained, but remain unclear. Results for the three intra-compartments are sparse, despite three regions being a main motivation for this work and were listed as advantage. Consider presenting these results more explicitly. Why not show confusion matrix for glycogen segmentation model?

**********

6. PLOS authors have the option to publish the peer review history of their article (what does this mean?). If published, this will include your full peer review and any attached files.

If you choose “no”, your identity will remain anonymous but your review may still be made public.

**Do you want your identity to be public for this peer review?**   For information about this choice, including consent withdrawal, please see our Privacy Policy.

Reviewer #1: No

Reviewer #2: No

Reviewer #3: No

**********

[NOTE: If reviewer comments were submitted as an attachment file, they will be attached to this email and accessible via the submission site. Please log into your account, locate the manuscript record, and check for the action link "View Attachments". If this link does not appear, there are no attachment files.]

To ensure your figures meet our technical requirements, please review our figure guidelines: https://journals.plos.org/plosone/s/figures

You may also use PLOS’s free figure tool, NAAS, to help you prepare publication quality figures: https://journals.plos.org/plosone/s/figures#loc-tools-for-figure-preparation.

NAAS will assess whether your figures meet our technical requirements by comparing each figure against our figure specifications.

# Author comment: Author response to Decision Letter 1

**DOI:** 10.1371/journal.pone.0351502.r002

16 May 2026

We thank the reviewers for their thoughtful comments and constructive suggestions. We have addressed all comments point-by-point below and believe that the revisions have improved the clarity and quality of the manuscript.

Reviewer #1: This is a very interesting and potentially useful methodological paper addressing an important barrier in the study of subcellular glycogen localisation in skeletal muscle. The topic is relevant, the manuscript is generally clear, and the open sharing of code, data, and model weights is a strength. However, I have several concerns that should be addressed before the manuscript can be considered further.

Overall, my main concern is that some of the conclusions appear stronger than the validation presented. The study shows promising agreement with manual image analysis, but there are important limitations related to the reference standard, the treatment of subsarcolemmal images, and the independence and size of the training and testing datasets.

Major comments

The manuscript presents the model as validated, robust, and objective. However, the reference standard appears to rely on manual annotation and point counting by a single experienced investigator. This demonstrates agreement with one expert, but not necessarily broader validity or reproducibility. Do authors have any data on how results differ between different experienced researchers?

Reply:

In this project, the model output was compared with analyses performed by a single experienced researcher (J.N.). The same researcher has led previous studies in which point-counting data from multiple researchers were compared. In these studies, inter-researcher bias reached up to 23% (e.g. Schytz et al., 2024). The bias observed for the model is therefore within the range of biases previously reported between experienced researchers.

We agree that the robustness of the model across different researchers has not been formally investigated. In the manuscript, the term “robust” was intended to refer to the model’s performance across different glycogen levels; however, we acknowledge that this was unclear in the abstract. Accordingly, we have added the following sentence (line 313-315):

“The level of bias (10–15%) is within the range of biases observed between different researchers (e.g. Schytz et al., 2024). We therefore interpret the model as a valid substitute for researcher-based manual analyses.”

In addition, we have removed the term “robust” from line 32.

A major concern is that the region model was trained on myofibrillar images, but then applied to subsarcolemmal images, with the intermyofibrillar class subsequently relabelled as subsarcolemmal. This appears to be an ad hoc solution and needs much stronger justification. If subsarcolemmal glycogen is a key part of the paper, this limitation should be more explicitly acknowledged and discussed.

Reply:

Due to the high magnification used, the image area was relatively small and contained predominantly subsarcolemmal glycogen. Therefore, during model development, we considered that the model’s ability to identify glycogen would likely be sufficient for estimating glycogen content within the subsarcolemmal region, despite not being specifically trained to distinguish subsarcolemmal from intermyofibrillar regions. The validation results support this assumption.

We agree with the reviewer that this limitation should be more explicitly acknowledged and discussed in the manuscript. Therefore, we have added the following paragraph to the Discussion (line 316-321):

“A limitation of the model is that it was not trained to discriminate between subsarcolemmal and intermyofibrillar regions. However, in most images classified as subsarcolemmal, contamination from the intermyofibrillar region was minimal due to the small image size (1.7 × 1.7 µm), in which the subsarcolemmal region occupied the majority of the image area. This may be specific to human muscle samples, and additional caution may therefore be required when applying the model to muscles with fewer subsarcolemmal organelles and inclusions.”

The rationale for the number of samples used for model development and evaluation should be explained more clearly. For example, in lines 108 to 116, images were obtained from 12 biopsies from 4 participants for manual segmentation and training, while all 21 biopsies were used for the biochemical comparison. Please explain how these numbers were determined and whether the authors believe that inclusion of more biopsies, fibres, or participants would further improve model performance or generalisability. This is particularly relevant for a machine learning based approach.

Reply:

The biopsies originated from a study including three time points with known large variability in glycogen content. These biopsies were therefore considered suitable for developing a model capable of handling a broad range of glycogen levels. To capture this variability, images were obtained from all three time points.

Initially, 429 images from two participants were manually annotated and used for model training. However, the resulting performance was considered insufficient for accurate estimation. Therefore, an additional 430 images from three other participants were annotated and included in the training dataset. No formal analysis was performed to determine how model performance scales with increasing numbers of biopsies, fibres, or participants, and we agree that inclusion of additional data could potentially further improve both model performance and generalisability.

To clarify this process, we have added information regarding these two training steps to the Methods section (lines 144–146): "For initial training 429 images from two participants were annotated but considered inadequate for accurate estimates. Therefore, additional 430 images from three other participants were annotated."

The biochemical comparison was included as a secondary validation in addition to the comparison with manually annotated ground truth data. Therefore, this analysis was performed using model predictions from all available biopsies (n = 21).

The results suggest systematic bias, with underestimation in intermyofibrillar and subsarcolemmal glycogen and overestimation in intramyofibrillar glycogen. This is an important limitation because the intended application of the model is compartment specific analysis. The discussion does acknowledge this, but I think the conclusion should be more cautious. Or, there should be some justified reassurance that this under/overestimation is small enough to still make the model valid.

Reply:

We agree that the observed systematic underestimation and overestimation across glycogen compartments represent an important consideration for compartment-specific analyses. However, we interpret these biases as sufficiently small to support the validity of the model, as they fall within the range of biases previously observed between experienced researchers performing manual analyses.

To clarify this justification, we have added the following statement to the Discussion (lines 313–315):

“The level of bias (10–15%) is within the range of biases observed between different researchers (e.g. Schytz et al., 2024). We therefore interpret the model as a valid substitute for researcher-based manual analyses.”

Last but not least - are authors confident that the presented model is sufficiently accurate to be used in further research by other scientists?

Reply:

We believe the presented model is sufficiently accurate to serve as a useful tool for future research applications. However, we also expect that optimal performance in new projects or datasets may require project-specific fine-tuning and validation.

To clarify this limitation, we have added the following statement to the Discussion (line 312):

“…and most likely a need for fine-tuning of the model.”

Minor comments

Line 49 - “Therefor” should be corrected to “therefore.”

Reply:

Done, thanks.

Lines 50 to 52 - It would help the reader if the authors briefly explained why analysis of 8 to 10 fibres per biopsy is typically needed. At present, this appears without much context. Since later results show that uncertainty decreases as more images are included, it would be useful to link this statement to the practical sampling considerations for biopsy level estimates.

Reply:

We agree that additional context was needed. There is currently no formal documentation establishing that 8–10 fibres are specifically required for reliable biopsy-level estimates. Rather, this number reflects a pragmatic balance between the time-consuming nature of the method and the well-known inter-fibre variability in glycogen content, where individual fibre values commonly vary within an approximately threefold range.

To clarify this point, we have modified the sentence in line 52 to read:

“…which is typically included in such studies.”

Lines 81 to 82 - Please provide a little more detail on what is meant by “samples were processed for transmission electron microscopy.” Even a brief summary of the key preparation steps would improve clarity for readers who are less familiar with TEM workflows, or a reference to a paper describing them in details.

Reply:

To clarify this, we now refer to the later section describing the TEM workflow. We have added (line 85): "(see fixation procedure below)".

Lines 84-92 - Authors state that two methods were used for biochemical method of glycogen quantification. However, it is unclear results of which method were subsequently used in the results section.

Reply:

We agree that this was unclear in the original manuscript. Therefore, we have added the following sentence (lines 97–101):

“The data used for comparison with TEM analyses were obtained using the Pentra C400 analyser for the pre-exercise and recovery samples, and the Fluoroskan instrument for the post-exercise samples. The Fluoroskan instrument has higher sensitivity for glycogen measurements at very low concentrations.”

Lines 90 to 92 Although this appears in the Methods, the phrase “unexpectedly low glycogen values” would benefit from a little more context. Unexpected relative to what, previous work, biological expectation, or the second assay? A short explanation would help readers understand why the second analytical approach was needed.

Reply:

We agree that additional context was needed. In fact, the term “unexpectedly low” was not appropriate, as the exercise protocol was identical to that used by Bergström and Hultman (1966), who reported similarly low glycogen levels. Therefore, we have replaced “unexpectedly low” with “very low.”

In addition, we have clarified why the second analytical approach was used by adding the following text (lines 97–101):

“The data used for comparison with TEM analyses were obtained using the Pentra C400 analyser for the pre-exercise and recovery samples, and the Fluoroskan instrument for the post-exercise samples. The Fluoroskan instrument has higher sensitivity for glycogen measurements at very low concentrations.”

Lines 108 to 116 - As noted above, please explain the reasoning behind the number of biopsies, participants, and fibres used for model training and validation. It would also be helpful to comment on whether larger training input might improve performance.

Reply:

The rationale for the two-step training procedure has now been clarified in lines 144–146: "For initial training 429 images from two participants were annotated but considered inadequate for accurate estimates. Therefore, additional 430 images from three other participants were annotated."

In addition, we have added the following explanation regarding the number of fibres analysed per biopsy (line 125-127):

“This number of fibres per biopsy has previously been shown to provide TEM-based estimates of total glycogen that are proportional to values obtained by biochemical determination (e.g. Schytz et al., 2024).”

We also acknowledge that increasing the number of biopsies, fibres, and participants included in the training dataset could potentially improve model performance and generalisability, although this was not formally investigated in the present study.

Reviewer #2: The authors developed a semantic segmentation model for glycogen particles in transmission electron microscopy images of skeletal muscle. Due to the time-consuming nature of manual analyses of glycogen particles and the critical role of glycogen in muscle metabolism, faster analytical methods are much needed. The developed model appears to work well. However, it would be helpful to better distinguish this model from a recently developed, similar glycogen model.

I appreciate that the authors are clear that their approach is similar to another recent work (Rios et al. J Gen Physiol 2024). However, it is not as clear why they developed a second approach instead of just adopting the original one. The authors state that the new model can better distinguish Z-disks and mitochondria than the previous model, and thus implied that this model is better equipped to assess fibers with different Z-disk widths and mitochondrial content. Demonstrating that the model performs well in different muscle fiber types would support these statements. Based on their extensive history of subcellular glycogen quantification, the authors likely have many ground truth images from different fiber types to compare with model output. Given that the code for the Rios et al. model appears to be freely available, and even better approach, but perhaps not necessary, would be to compare the current model versus the original model.

Reply:

The development of our model was initiated before the code from the study by Eduardo Ríos et al. became publicly available. Once the code was released, we contacted Eduardo Ríos to discuss potential directions for the project.

A key objective of our model development was the ability to distinguish between Z-disks and mitochondria, as this would enable future studies investigating glycogen localization in relation to mitochondrial volume fraction. For this reason, we continued the development of our own model rather than directly adopting the existing approach.

We agree that demonstrating performance across different muscle fibre types would strengthen this point. However, in our previous studies, glycogen content was quantified using point-counting methods to estimate glycogen areal fractions, and therefore no pixel-level ground truth annotations are available from those datasets for direct validation of the present model.

Reviewer #3 raised a similar question regarding comparison with the model by Rios et al., and we therefore refer to our response to Reviewer #3 for additional details on this point.

Since the major error seems to be in region segmentation rather than glycogen segmentation, it would be helpful to see the ground truth segmentations included in the panels in Figures 1 and 2.

Reply:

The ground truth has been added to Figures 1 and 2.

Figure 1: What do the different colors represent in the Predicted Glycogen panels? It is not clear from the figure, legend, or text. If it is glycogen probabilities as it looks to be, it would be helpful to also include the binary glycogen images (using probability threshold of 0.3 as stated in methods) to make it clear which glycogen particles are counted and which are not.

Reply:

We agree. We have changed the predictions to be the binary glycogen images at threshold 0.3.

Minor Comments

Methods, Participants and Muscle Samples: Which biopsy method was used?

Reply:

Added to line 82: "(using the Bergström needle with suction)"

Reviewer #3: The paper tackles a challenging problem with a well-motivated approach. However, the clarity of the presentation needs improvement, making it difficult to fully appreciate the paper's contributions.

Bellow, I list specific points that could be addressed to enhance the clarity and impact of the paper.

The relationship to the parallel approach of Rios et al. is unclear. Lines 55-62 attempt to address this, but the wording is vague. The use of past tense ("we aimed to develop") and the mention of Rios's model being available raise doubts about whether your model was developed as intended. It i

Attachment Submitted filename: Response to Reviewers.docx

# Peer review: Decision Letter 1

**DOI:** 10.1371/journal.pone.0351502.r003

**Authors:** Everson Nunes

28 May 2026

A semantic segmentation model to predict subcellular glycogen localization using transmission electron microscopy images

PONE-D-26-06780R1

Dear Dr. Nielsen,

We’re pleased to inform you that your manuscript has been judged scientifically suitable for publication and will be formally accepted for publication once it meets all outstanding technical requirements.

Within one week, you’ll receive an e-mail detailing the required amendments. When these have been addressed, you’ll receive a formal acceptance letter and your manuscript will be scheduled for publication.

An invoice will be generated when your article is formally accepted. Please note, if your institution has a publishing partnership with PLOS and your article meets the relevant criteria, all or part of your publication costs will be covered. Please make sure your user information is up-to-date by logging into Editorial Manager at Editorial Manager® and clicking the ‘Update My Information' link at the top of the page. For questions related to billing, please contact billing support.

If your institution or institutions have a press office, please notify them about your upcoming paper to help maximize its impact. If they’ll be preparing press materials, please inform our press team as soon as possible -- no later than 48 hours after receiving the formal acceptance. Your manuscript will remain under strict press embargo until 2 pm Eastern Time on the date of publication. For more information, please contact onepress@plos.org.

Kind regards,

Everson Nunes, Ph.D.

Academic Editor

PLOS One

Reviewers' comments:

Reviewer's Responses to Questions

**Comments to the Author**

1. If the authors have adequately addressed your comments raised in a previous round of review and you feel that this manuscript is now acceptable for publication, you may indicate that here to bypass the “Comments to the Author” section, enter your conflict of interest statement in the “Confidential to Editor” section, and submit your "Accept" recommendation.

Reviewer #1: All comments have been addressed

Reviewer #2: All comments have been addressed

**********

2. Is the manuscript technically sound, and do the data support the conclusions?

The manuscript must describe a technically sound piece of scientific research with data that supports the conclusions. Experiments must have been conducted rigorously, with appropriate controls, replication, and sample sizes. The conclusions must be drawn appropriately based on the data presented.

Reviewer #1: Yes

Reviewer #2: Yes

**********

3. Has the statistical analysis been performed appropriately and rigorously?

Reviewer #1: Yes

Reviewer #2: Yes

**********

4. Have the authors made all data underlying the findings in their manuscript fully available?

The PLOS Data policy requires authors to make all data underlying the findings described in their manuscript fully available without restriction, with rare exception (please refer to the Data Availability Statement in the manuscript PDF file). The data should be provided as part of the manuscript or its supporting information, or deposited to a public repository. For example, in addition to summary statistics, the data points behind means, medians and variance measures should be available. If there are restrictions on publicly sharing data—e.g. participant privacy or use of data from a third party—those must be specified.

Reviewer #1: Yes

Reviewer #2: Yes

**********

5. Is the manuscript presented in an intelligible fashion and written in standard English?

PLOS ONE does not copyedit accepted manuscripts, so the language in submitted articles must be clear, correct, and unambiguous. Any typographical or grammatical errors should be corrected at revision, so please note any specific errors here.

Reviewer #1: Yes

Reviewer #2: Yes

**********

6. Review Comments to the Author

Please use the space provided to explain your answers to the questions above. You may also include additional comments for the author, including concerns about dual publication, research ethics, or publication ethics. (Please upload your review as an attachment if it exceeds 20,000 characters)

Reviewer #1: I have no further comments and I am looking forward to seeing more publications assessing the localisation of glycogen within skeletal muscle as a result of simplified analysis method.

Reviewer #2: (No Response)

**********

7. PLOS authors have the option to publish the peer review history of their article (what does this mean?). If published, this will include your full peer review and any attached files.

If you choose “no”, your identity will remain anonymous but your review may still be made public.

**Do you want your identity to be public for this peer review?**   For information about this choice, including consent withdrawal, please see our Privacy Policy.

Reviewer #1: No

Reviewer #2: **Yes:**   Brian Glancy

**********

# Editor report: Acceptance letter

**DOI:** 10.1371/journal.pone.0351502.r004

**Authors:** Everson Nunes

PONE-D-26-06780R1

PLOS One

Dear Dr. Nielsen,

I'm pleased to inform you that your manuscript has been deemed suitable for publication in PLOS One. Congratulations! Your manuscript is now being handed over to our production team.

At this stage, our production department will prepare your paper for publication. This includes ensuring the following:

* All references, tables, and figures are properly cited

* All relevant supporting information is included in the manuscript submission,

* There are no issues that prevent the paper from being properly typeset

You will receive further instructions from the production team, including instructions on how to review your proof when it is ready. Please keep in mind that we are working through a large volume of accepted articles, so please give us a few days to review your paper and let you know the next and final steps.

Lastly, if your institution or institutions have a press office, please let them know about your upcoming paper now to help maximize its impact. If they'll be preparing press materials, please inform our press team within the next 48 hours. Your manuscript will remain under strict press embargo until 2 pm Eastern Time on the date of publication. For more information, please contact onepress@plos.org.

You will receive an invoice from PLOS for your publication fee after your manuscript has reached the completed accept phase. If you receive an email requesting payment before acceptance or for any other service, this may be a phishing scheme. Learn how to identify phishing emails and protect your accounts at https://explore.plos.org/phishing.

If we can help with anything else, please email us at customercare@plos.org.

Thank you for submitting your work to PLOS ONE and supporting open access.

Kind regards,

PLOS ONE Editorial Office Staff

on behalf of

Dr. Everson Nunes

Academic Editor

PLOS One
